# Optimizing the integration of family caregivers in the delivery of person-centered care: evaluation of an educational program for the healthcare workforce

**DOI:** 10.1186/s12913-022-07689-w

**Published:** 2022-03-18

**Authors:** Jasneet K. Parmar, Tanya L’Heureux, Sharon Anderson, Wendy Duggleby, Cheryl Pollard, Lisa Poole, Lesley Charles, Lyn K. Sonnenberg, Myles Leslie, Gwen McGhan, Arlene Huhn, Sandy Sereda, Cecilia Marion, Glenda Tarnowski, Jennifer Mah, Denise Melenberg, Carolyn Weir, Charlotte Pooler, Nora MacLachlan, Suzette Bremault-Phillips, Peter George J. Tian, Lori-Ann R. Sacrey

**Affiliations:** 1 Department of Family Medicine, Faculty of Medicine and Dentistry, University of Alberta, 6-40 University Terrace, 8303 112 St NW, Edmonton, AB T6G 2T4 Canada; 2 Home Living and Transitions, Alberta Health Services – Edmonton Zone Continuing Care, Strathcona Health Centre, 2 Brower Drive, Baseline Road, Sherwood Park, AB T8H1V4 Canada; 3 Department of Human Ecology, University of Alberta, 335 Human Ecology Building, 8905 - 116 St NW, Edmonton, Alberta T6G 2N1 Canada; 4 Faculty of Nursing, University of Alberta, 3-141 Edmonton Clinic Health Academy (ECHA), Edmonton, Alberta T6G 1C9 Canada; 5 Faculty of Nursing, University of Regina, RI 508, 3737 Wascana Parkway, Regina, Saskatchewan S4S 0A2 Canada; 6 Dementia Advocacy Canada, Calgary Dementia Network Strategic Council, Glencoe Club Dementia Advisory Group, Calgary, Canada; 7 Care of the Elderly, Glenrose Rehabilitation Hospital-East, 10230 111 Ave NW, Edmonton, AB T5G 0B7 Canada; 8 Faculty of Rehabilitation Medicine, University of Alberta, 3-14 Corbett Hall, Edmonton, AB T6G 2G4 Canada; 9 School of Public Policy, University of Calgary, Calgary, 906 8th Avenue S.W. 5th Floor, Alberta T2P 1H9 Canada; 10 Department of Community Health Sciences, Cumming School of Medicine, University of Calgary, Calgary, 3D10, 3280 Hospital Drive NW, Alberta T2N 4Z6 Canada; 11 Faculty of Nursing, University of Calgary, PF 3204, 2500 University Drive NW, Calgary, AB T2N 1N4 Canada; 12 Client Services & Programs, Alzheimer Society of Alberta and Northwest Territories, Suite 306, 10430-61 Avenue, Edmonton, AB T6H 2J3 Canada; 13 Caregivers Alberta, 12122 68 St NW, Edmonton, Alberta T5B 1R1 Canada; 14 Youville Home, Covenant Health, 9A St Vital Ave, St. Albert, Alberta T8N 1K1 Canada; 15 College of Licensed Practical Nurses of Alberta, St. Albert Trail Place, 13163-146 Street, Edmonton, AB T5L 4S8 Canada; 16 Faculty of Health and Community Studies, Norquest College, Edmonton, Canada; 17 Palliative Care Programs/Education and Practice Development/Community Programs, Alberta Health Services, Edmonton, Canada; 18 Mother Rosalie Health Service Centre, 16930 87 Ave, Edmonton, AB T5R4H5 Canada; 19 Health and Community Studies, Bow Valley College, Calgary, North Campus, Seventh Floor, 345 - 6 Avenue SE., Alberta AB T2G 4S6 Canada; 20 Division of Care of the Elderly, Department of Family Medicine, University of Alberta, 6-40 University Terrace, 8303 112 St NW, Edmonton, AB T6G 2T4 Canada; 21 Department of Pediatrics, Faculty of Medicine and Dentistry, University of Alberta, Edmonton Clinic Health Academy, 11405-87 Avenue, Edmonton, Alberta T6G 1C9 Canada; 22 Autism Research Centre (E209), Glenrose Rehabilitation Hospital, 10230 - 111 Avenue, Edmonton, AB T5G 0B7 Canada

**Keywords:** Person-centered, Health workforce education, Family caregivers, Carer, Kirkpatrick-Barr

## Abstract

**Background:**

While family caregivers provide 70-90% of care for people living in the community and assist with 10-30% of the care in congregate living, most healthcare providers do not meaningfully involve family caregivers as partners in care. Recent research recommends that the healthcare workforce receive competency-based education to identify, assess, support, and partner with family caregivers across the care trajectory.

**Objective:**

This paper reports a mixed-methods evaluation of a person-centered competency-based education program on Caregiver-Centered Care for the healthcare workforce.

**Methods:**

This foundational education was designed for all healthcare providers and trainees who work with family caregivers and is offered free online (caregivercare.ca). Healthcare providers from five healthcare settings (primary, acute, home, supportive living, long-term care) and trainees in medicine, nursing, and allied health were recruited via email and social media. We used the Kirkpatrick-Barr health workforce training evaluation framework to evaluate the education program, measuring various healthcare providers’ learner satisfaction with the content (Level 1), pre-post changes in knowledge and confidence when working with family caregivers (Level 2), and changes in behaviors in practice (Level 3).

**Results:**

Participants were primarily healthcare employees (68.9%) and trainees (21.7%) and represented 5 healthcare settings. Evaluation of the first 161 learners completing the program indicated that on a 5-point Likert scale, the majority were satisfied with the overall quality of the education (Mean(M) = 4.69; SD = .60). Paired T-tests indicated that out of a score of 50, post-education changes in knowledge and confidence to work with family caregivers was significantly higher than pre-education scores (pre M = 38.90, SD = 6.90; post M = 46.60, SD = 4.10; *t*(150) = − 16.75, *p* < .0001). Qualitative results derived from open responses echoed the quantitative findings in satisfaction with the education delivery as well as improvements in learners’ knowledge and confidence.

**Conclusion:**

Health workforce education to provide person-centered care to all family caregivers is an innovative approach to addressing the current inconsistent system of supports for family caregivers. The education program evaluated here was effective at increasing self-reported knowledge and confidence to work with family caregivers.

**Supplementary Information:**

The online version contains supplementary material available at 10.1186/s12913-022-07689-w.

Worldwide, family caregivers [FCGs] are the backbone of long-term care for people with physical and mental illness, disabilities, and frailty due to aging [1–3]. FCGs provide 70-90% of care to people living in community homes who need care [4–6] and, before the COVID-19 pandemic, were assisting with approximately 30% of the care in congregate care (e.g., supportive and assisted living, long-term care) [7, 8]. Moreover, an aging population, longer life expectancies, and better medical care have increased the demand for FCGs, as well as the length of the care trajectory [1, 9–11]. While caregiving responsibilities typically change and grow as the care receiver’s needs increase [12–14], the care provided by FCGs has also increased with respect to the complexity of care tasks and the care intensity [1–3]. In the United Kingdom, Buckner and Yeandle [1] report that the proportions of FCGs providing 20-49 h per week increased by 42% and those providing 50 or more hours per week rose by almost 33% between 2001 and 2015. The increased workload of FCGs of home care clients in Canada has also been reported to be as high as an average of 115 h of care per week [15]. In addition to hands-on care work, FCGs are now spending 15-50% of their time navigating, negotiating, and managing services within health and social care systems [1, 13, 14].

Although 88% of FCGs who care for older parents report that caregiving can be rewarding [16], being overwhelmed with care work and worry can have substantial impacts on their own mental and physical health [1–3]. The caregivers at highest risk of burden [17, 18], anxiety [19, 20], and loneliness [17, 21] are those who provide more than 20 h of care per week, perform medical/nursing care tasks, are involved in complex decision making, and/or care for a receiver who resides with them, has significant physical disabilities, and/or has depression, dementia, or responsive behaviors [13, 22, 23].

In the last 20 years, a plethora of research studies and reports have recommended that healthcare providers -- a group that includes physicians, nurse practitioners, physician assistants, nurses, social workers, psychologists, pharmacists, allied health, healthcare aides, certified nursing assistants, physician assistants, and others – who work with FCGs should have the competencies to recognize FCGs’ roles and contributions, engage them as partners in care, support their ability to maintain their own wellbeing, and assist them to navigate available services and supports [1, 11, 13, 24–26]. Despite this recommendation, FCGs are marginalized by the healthcare system [13, 27].

Imperatives for healthcare providers to support FCGs have come primarily from caregiver advocacy organizations or from FCGs rather than from healthcare providers [28, 29]. While healthcare providers acknowledge FCGs would benefit from support [29–33], there are many reasons why healthcare providers/systems have not systematically included FCGs as partners on the care team, systematically assessed or addressed the FCG’s needs, or helped them to navigate the health and social care systems. Healthcare providers may not see supporting FCGs as within their role or part of basic care [29], citing an ethical responsibility to the patient [34, 35], and thus concerns with patient autonomy and privacy [35, 36] as well as lack of time, knowledge, and reimbursement for caregiving issues which can be complex, emotionally draining and time-consuming [28, 36, 37].

To address these -gaps in care and support for FCGs, multidisciplinary stakeholders in Alberta identified the person-centered competency domains and indicators [38] that healthcare providers require to work with diverse FCGs. Education to prepare healthcare providers to identify, engage and partner with, assess their support needs as well as willingness and ability to provide care, and support FCGs to sustain care and maintain their own wellbeing is an innovative approach to addressing an inconsistent system of supports for FCGs [13, 29, 39]. Moreover, healthcare workplace education should reflect the broader needs of the healthcare system [40]. Healthcare providers are ideally positioned to engage FCGs as partners in care, as well as to support them to maintain their own health and wellbeing [41, 42]. Caregiver diversity and the diverse nature of their caregiving situations calls for person-centered supports [13, 42, 43]. There are many definitions of person-centered care and all embrace ensuring that people are involved in, and central to their own care [44, 45]. The person receiving care and their families define their “family” or FCG and determine how they will participate in care and decision-making [46]. Person-centered, patient-centered, or family-centered care are associated with care that includes:

- involving people in decision making that respects their values, needs, and preferences;
- customizing communication, information, and education to the individual’s needs;
- providing emotional support to reduce the person’s anxiety and treatment fears;
- coordinating and integrating care to alleviate people’s feelings of powerlessness and vulnerability;
- ensuring care is accessible when it is needed [45–47].

While providing person-centered care is a key goal for health systems [48, 49], the term “family caregiver” or “carer” is not currently associated with definitions of person-centered or person- and family-centered care. Thus, the need for person-centered care for FCGs may not be formally recognized by providers within health and social systems [50, 51]. To ensure a specific person-centered focus on FCGs, we created the term “Caregiver-Centered Care,” defined as *“a collaborative working relationship between families and healthcare providers in supporting FCGs to maintain their own wellbeing and in their caregiving role, decisions about services, care management, and advocacy”* [52]. We emphasize that person-centered care for FCGs does not reduce the emphasis on care for the patient, nor does it mean shifting care responsibility, management, or advocacy to the family caregiver. Rather, it is a collaborative working relationship of the healthcare workforce, working with and supporting FCGs in their caregiving role which further enhances the patient’s quality of life and increases efficient use of health and community resources [13, 43].

The Caregiver-Centered Care Competency Framework was validated in a Modified Delphi process [38]. We used these competencies and a literature review of evidence-based [53, 54] and best practices [55–57] to inform the design of the Foundational Caregiver-Centered Care Education for all healthcare providers who interact with FCGs. In this article, we report on the mixed methods evaluation of the Foundational Caregiver-Centered Care Education. To date, we have not found any other published research evaluations of health workforce education that provides person-centered care for FCGs.

## Methods

### The foundational caregiver-centered care education

The Foundational Caregiver-Centered Care Education program was co-designed by over 100 multi-level, interdisciplinary stakeholders including policy makers, researchers, health care administrators and providers, educators, not-for-profit community leaders, and FCGs [58]. We utilized adult learning theories including constructivism and transformative learning theory, which view learners as active participants in constructing knowledge and meaning through critical reflection upon new information and their experiences [59, 60]. We also drew upon best practices in health workforce education [55, 61] to inform education design. The education consists of six modules that follow the domains in competency framework [28, 42], including (a) Recognizing the FCG Role, (b) Communicating with FCGs, (c) Partnering with FCGs, (d) Fostering Resilience in FCGs, (e) Navigating Health and Social Systems and Accessing Resources, and (f) Enhancing the Culture and Context of Healthcare. The teaching and learning resources include six videos interspersed with interactive exercises designed to encourage learners to reflect on how the learning might be useful in their role and setting (See Supplementary Materials 1) Key learning points and 2) Interactive exercises). The education was designed to be delivered flexibly, either facilitated-in-person or virtually. Due to the Covid-19 pandemic preventing in-person learning opportunities, the education is offered free online (caregivercare.ca) and takes about an hour to complete. Participants receive a certificate on completion.

### Evaluation study design

**Fig. 1:**
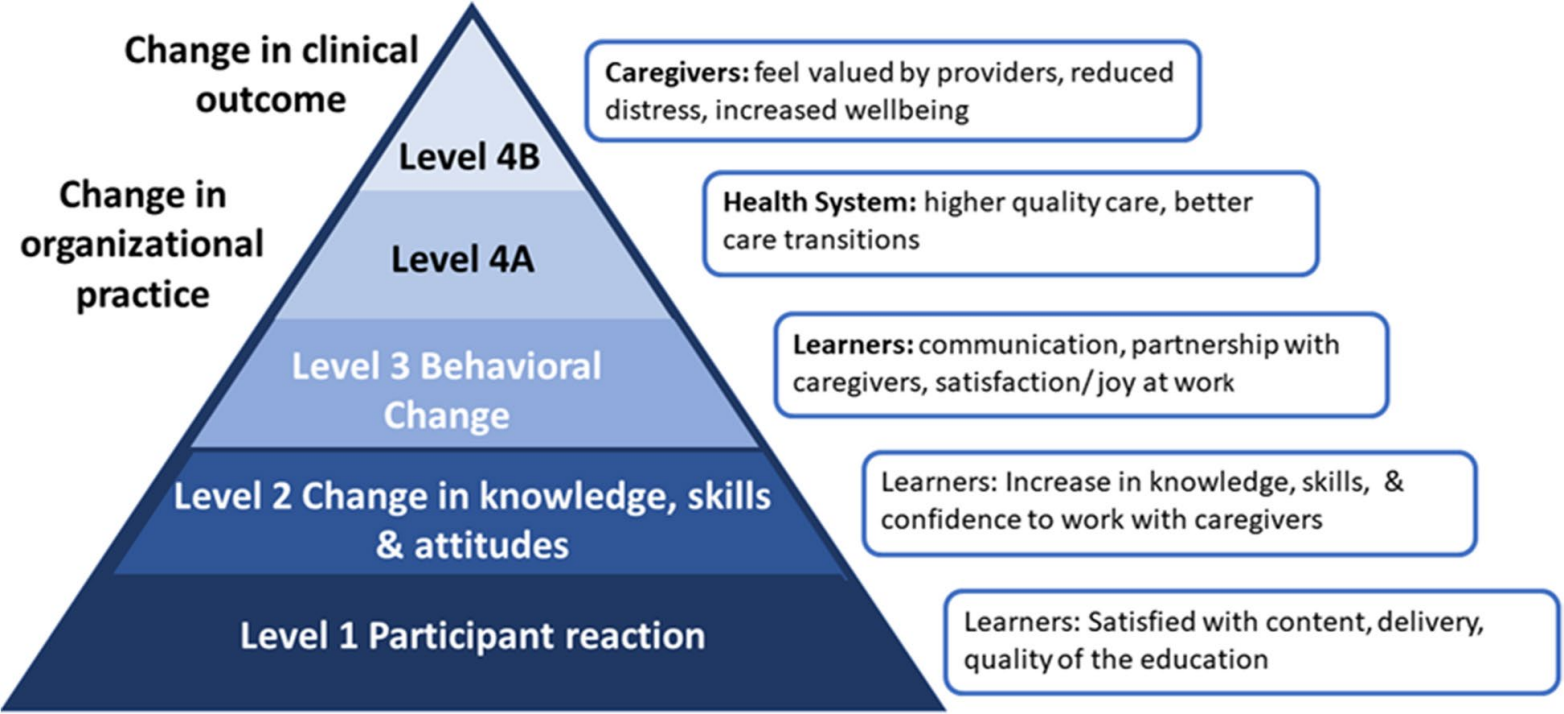
Impacts of Caregiver-Centered Care Mapped to Kirkpatrick-Barr Framework adapted from [68, 69]

We followed the development and validation of the guideline for reporting evidence-based practice educational interventions and teaching (GREET) in this paper [62]. We undertook a within subject pre-post-test mixed methods triangulation design evaluation [63, 64] informed by the Kirkpatrick-Barr health workforce education evaluation framework [65, 66]. The objective of using mixed methods triangulation is to obtain distinct but complementary data [63, 64]. This can be classified as a type of convergent parallel mixed-method design, which engages in comprehensive analysis through integrating quantitative and qualitative data [64, 67]. The Kirkpatrick framework proposes evaluating training effectiveness at four levels: Level 1 Participant reaction refers to participant’s satisfaction with the educational program and delivery; Level 2 Change in knowledge, skills or attitudes involves participant’s knowledge acquisition to change skills, attitudes, or confidence; Level 3 Behavioural Change refers to changes in participant’s behaviors as the result of the program; Level 4a Change in organizational practice entails wider change in organizational practice and delivery of care; and Level 4b Change in clinical outcome refers to the impacts of the educational program on patients and caregivers [68, 69]. In this report, we focus on our evaluations of Levels 1, 2, and 3 (see Fig. 1 adapted from [68, 69]). The study and all data collection tools were approved by the Health Research Ethics Board at the University of Alberta (Study ID Pro00097068). All methods were performed in accordance with the relevant ethics guidelines and methods. Healthcare providers from five healthcare settings (primary, acute, home, supportive living, long-term care) and trainees in medicine, nursing, and allied health were recruited via emails from healthcare managers or educators and social media posts (Twitter, Linked-in, Facebook). All participants provided informed implied consent by clicking continue to the education after reading the ethics information letter. They were free to leave the study at any time.

### Data collection

We collected the following data online through Google survey software and Google sheets data collection tools: (1) participant characteristics, (2) learner’s reactions to the education, and (3) pre- post changes in knowledge and confidence to work with FCGs.

### Participant characteristics

We collected demographic data on participant sex, age, employment setting (employed in healthcare, trainee, employed in social care, FCG, other), occupation, care setting (acute, social, home, long-term, primary, supportive living, trainee), and province.

### Learner’s reactions to the education (level 1)

We used six questions to measure learner’s reactions to the education (Level 1). Five quantitative questions measured learner’s satisfaction, “The goals of this education were clear”; “Overall, the quality of the course content was excellent”;” The videos helped to increase my understanding of FCGs”; and “The exercises between the videos increased my knowledge”; and” I am motivated to learn more about Caregiver-Centered-Care.” Learners provided ratings on a five-point Likert Scale (strongly disagree to strongly agree). We included one qualitative question, “Please use the space below to provide any further comments about the Caregiver-Centered Care Education.”

### Pre-post changes in learner’s knowledge and confidence (level 2)

It is critical for credible evaluation that the measurement quality aligns with best practice for reliability and validity [55, 56]. We used the Caregiver-Centered Care Competency Framework [38] to design the ten question Caregiver-Centered Care Knowledge Assessment Test [CKAT]. See Table 1: Caregiver-Centered Care Knowledge Assessment Test. The Caregiver-Centered Care Competency Framework, was created in consultation with international, national, and provincial multi-level interdisciplinary stakeholders and validated in a Modified Delphi process [24, 38]. The CKAT was constructed to assess learners self-rated pre- and post-education changes in their knowledge and confidence to work with FCGs (Level 2). Learners were asked to rate their level of agreement to the ten statements on a 5-point Likert Scale, from strongly disagree to strongly agree.

**Table 1:** Caregiver-Centered Care Knowledge Assessment Test (CKAT)

***Please indicate your level of agreement to the following questions.***	
1. I am aware of the contributions of family caregivers.	
2. I am aware of the consequences of caring to family caregivers.	
3. I am comfortable in identifying family caregivers.	
4. I know how to communicate with family caregivers.	
5. I know what it means to partner with family caregivers.	
6. I know my role in assessing caregiver needs.	
7. I know how to assist family caregivers to navigate the system.	
8. I am confident in my knowledge to support family caregivers.	
9. I am comfortable in supporting family caregivers.	
10. I understand the need to reflect on my interactions with family caregivers.	

The first draft of the CKAT was reviewed by members of the research team with expertise in health workforce education and family caregiving. They scrutinized question clarity and ensured the questions were related to the content of the education. This resulted in minor changes in the structure or wording of four of ten questions. We pilot tested the questions with a convenience sample of healthcare providers (*n* = 20) employed in continuing care. Participants took 5 to 10 min to complete the questions. We calculated for Cronbach’s alpha to determine internal reliability of the pilot questionnaire. The Cronbach’s alpha was 0.85 for pre-test and 0.83 for post-test. In the current evaluation, the CKAT Cronbach alpha co-efficient was 0.92 for pre-test and 0.93 for post-test.

We used factor analysis to further assess the dimensionality of the CKAT [70, 71]. We wanted to know if there was a difference in the factors measuring changes in knowledge and confidence. We assessed the suitability of the data for factor analysis prior to performing the principal component analysis. All of the co-efficients in the correlation were above .30 (range = .41 to .76) [70, 72]. The Kaiser-Meyer-Olkin value was .91 (exceeding the recommended value of .60), indicating the sampling was adequate [71]. Bartlett’s Test of Sphericity was statically significant (*p* < .001), which supports the factorability of the correlation matrix [70, 71]. Two factors had eigenvalues exceeding Kaiser’s criterion of 1, explaining 68.2% of the variance (38.7 and 29.5%, respectively). The scree plot was ambiguous and show inflections that could justify one or two factors. The factor matrix supported a single factor (See Supplementary material 3: Factor Matrix).

### Behavioral change from education (level 3)

Semi-structured interviews with learners 6 months after they had taken the education were used to understand how learners had used the education in practice (See Supplementary material 4: Semi-structured Interviews Level 3). We conducted interviews on ZOOM with 13 people who completed the education to understand learners’ perceptions of how the education had influenced their interactions with FCGs. To ensure participants from a range of time points (early participants to 6 months post-launch), we selected every 5th participant who completed the education who had consented to be contacted for a follow-up interview. The strategy resulted in a variety of students as well as new and seasoned providers from five professions (nurses, allied health, social work, physician, healthcare aide) partaking in qualitative interviews.

### Data analysis

Quantitative data were analyzed using SPSS® version 27 [73]. Proportions were calculated for categorical variables and sample means and standard deviations were calculated for individual scale items and total scale scores. We used Student’s paired T-tests to examine the differences in pre-test and post-test CKAT scores. All *p* values < 0.05 were considered significant. We compared learner’s qualitative reports with quantitative results.

Qualitative data were analyzed thematically [74]. Thematic analysis is a flexible qualitative method used to explore the different perspectives held by research participants; it highlights the similarities and divergences in their viewpoints and generates thematic insights [75]. We methodically followed Braun and Clarke’s [74, 75] six stages of analysis (see Supplementary Material 5: Stages of Thematic Analysis).

Two members of the research team independently read through the 1) qualitative quotes and 2) interview transcripts while listening to the digitally recorded interviews. To become familiar with the data and to generate first impressions of meaning (stage one), they made notes of their impressions on MS Word transcripts. They discussed the initial impressions, then imported the data into NVivo® [76]. In stage two, members of the research team worked separately to inductively generate initial open codes. In stage three, team members worked together to generate categories. Patterns within the open codes were identified and codes with similar attributes and meanings were grouped. The categories were then refined into preliminary themes (stage four). At stage four, we discussed how healthcare providers applied their knowledge gained from the education in their work with FCGs. We then reread the transcripts to name and confirm the final themes (stage five). The report was generated (stage six) and discussed at a final team meeting.

## Results

We collected linked pre- and post- CKAT data during a 2-month time period in which 161 people completed the evaluation. Only data of participants with complete pre-post data on all 10 questions (*n* = 150; 93%) were included in the analysis.

### Participant characteristics

As shown in Table 2, almost two-thirds of the sample were female (62.7%), age grouping was well distributed, with each group of learners younger than 65 years of age comprising approximately 20% per age group. Participants were primarily healthcare employees (68.1%), healthcare students (21.4%), and employees in community social care (4.5%), who worked in acute care (14.9%), primary care (11.2%), home care (21.7%), supportive living (3.7%) and long-term care (8.1%). While all healthcare professions were represented, more than a third (33.7%) were nurses (nurse practitioners, registered nurses, licensed practical nurses). See Table 2 Characteristics of participants.

**Table 2:** Characteristics of participants

	Frequency	Percent
Sex		
Male	59	36.6%
Female	101	62.7%
Other	0	0.0%
Not answered	1	0.6%
Total	161	100.0%
Age		
≤ 24 years	29	18.0%
25–34	41	25.5%
35–44	25	15.5%
45–54	37	23.0%
55–64	26	16.1%
65+	3	1.9%
Not answered	0	0.0%
Total	161	1
Employment setting		
Employed in healthcare	111	68.9%
Trainee	35	21.7%
Caregiver	1	0.6%
Employed in Social/Community Care	11	6.8%
Other	3	1.9%
Not answered	0	0.0%
Total	161	1
Work Setting		
Acute Care	24	14.9%
Primary Care	18	11.2%
Homecare	35	21.7%
Supportive Living	6	3.7%
Long-term care	13	8.1%
Community Social Care	11	6.8%
Student or trainee	35	21.7%
Educator/ policymaker/other	4	2.5%
Not answered	15	9.3%
Total	161	1
Occupation		
Licensed Practical Nurse	29	18.0%
Registered Nurse	28	17.4%
Rec Therapist	14	8.7%
Allied Health (OT, PT, SLP)	8	5.0%
Health care aide	8	5.0%
Radiation therapist	8	5.0%
Physician	6	3.7%
Social Worker	3	1.9%
Nurse Practitioner	3	1.9%
Administration	3	1.9%
Caregiver	1	0.6%
Student or trainee	35	21.7%
Not answered	15	9.3%
Total	161	100.0%
Canadian Province		
AB	104	64.6%
BC	1	0.6%
SK	5	3.1%
MB	1	0.6%
ON	44	27.3%
PQ	3	1.9%
Outside Canada	3	1.9%
Total	161	1

### Learners’ reaction: satisfaction with the education (level 1)

Generally, learners were satisfied with the education. On the 5-point Likert scale, means of the five questions related to satisfaction with the education ranged from 4.5 (exercises between the videos increased my knowledge) to 4.8 (goals were clear and videos increased understanding of FCGs). See Table 3 for descriptive statistics of learner’s ratings of their satisfaction with the education.

**Table 3:** Learners’ reaction, satisfaction with the education (Level 1)

	n	Minimum	Maximum	Median	Mean	Std. Deviation
The goals of this education were clear.	160	3	5	5	4.8	0.49
Overall, the quality of the course content was excellent.	159	2	5	5	4.8	0.57
The videos content helped to increase my understanding of family caregivers.	159	2	5	5	4.8	0.56
The exercises between the videos increased my knowledge.	159	2	5	5	4.5	0.73
I am motivated to learn more about Caregiver-Centered-Care.	159	2	5	5	4.7	0.55

The positive qualitative comments in the open-ended question in the surveys reflected the high quantitative scores.

*This module has increased the confidence I have with regards to caregiving centered care. The key points that were discussed on this module were so clear and precise and I would highly recommend for my colleagues to take this course as well. (Occupational therapist)*

*I loved this course because it showed various professionals in the healthcare setting. The videos weren't fake looking. I could actually believe the story you were telling. As a future social worker and current homecare staff I am very thankful for the course and I know I will utilize these steps. It has given me a solid foundation to build more knowledge since you don't know what you don't know! (Social work student)*

The principal recommendation in response to one the qualitative question was to add closed captioning to the videos, which we added after the evaluation was completed.

### Changes in learner’s knowledge and confidence (level 2)

**Fig. 2:**
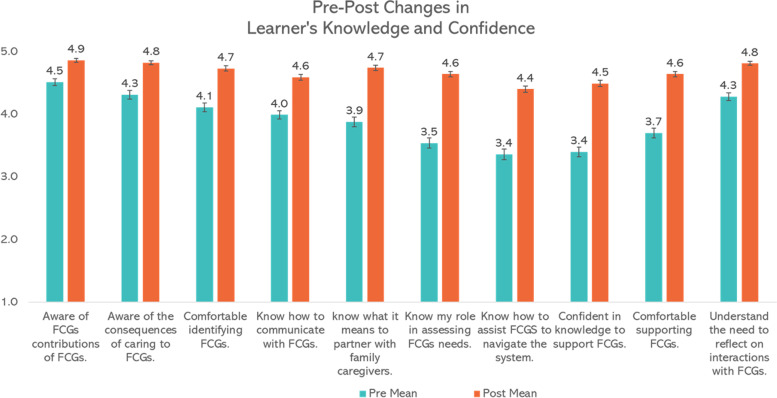
Kirkpatrick Level 2: Pre-Post Changes in Learner’s Knowledge and Confidence (With Standard Error Bars)

We obtained complete pre- and post- quantitative data from 150 of the 161 people who first completed the education. Within-person students’ paired T-tests indicated pre-post changes in learner’s knowledge and confidence to work with FCGs were significant for the 5-point Likert scale on all ten questions and for the total scale score out of 50 (Pre [M = 38.90, SD = 6.90] to post [M = 46.60, SD = 4.10]; *t*(150) = − 16.75, *p* < .0001 [two-tailed]). See Table 4 and Fig. 2. The differences between sub-groups of professions (e.g., physicians/nurses allied health/nurses) or workplace setting were not significant.

**Table 4:** Pre-post changes in learners’ knowledge and confidence (level 2)

		Paired Differences					t	df	Sig. (2-tailed)
		Mean	Std. Deviation	Std. Error Mean	95% Confidence Interval of the Difference				
					Lower	Upper			
Total Scale Score (5–50).	Pre-post total	−7.69	5.65	0.46	−8.60	−6.78	− 16.75	150	< 0.001
I am aware of the contributions of family caregivers.	Pre1 - Post1	−0.36	0.63	0.05	− 0.45	− 0.26	−7.18	159	< 0.001
I am aware of the consequences of caring to family caregivers.	Pre2 - Post2	−0.52	0.76	0.06	−0.64	−0.39	−8.54	158	< 0.001
I am comfortable in identifying family caregivers.	Pre3 - Post3	−0.62	0.78	0.06	−0.74	−0.50	−10.11	158	< 0.001
I know how to communicate with family caregivers.	Pre4 - Post4	−0.60	0.75	0.06	−0.72	−0.48	−10.03	155	< 0.001
I know what it means to partner with family caregivers.	Pre5 - Post5	−0.86	0.92	0.07	−1.01	−0.71	− 11.68	156	< 0.001
I know my role in assessing caregiver needs.	Pre6 - Post6	−1.1	0.90	0.07	−1.24	−0.96	−15.48	159	< 0.001
I know how to assist family caregivers to navigate the system.	Pre7 - Post7	−1.04	0.99	0.08	−1.19	−0.88	−13.18	157	< 0.001
I am confident in my knowledge to support family caregivers.	Pre8 - Post8	−1.09	0.92	0.07	−1.24	−0.95	−15.09	159	< 0.001
I am comfortable in supporting family caregivers.	Pre9 - Post9	−0.94	0.89	0.07	−1.083	−0.804	−13.38	159	< 0.001
I understand the need to reflect on my interactions with family caregivers.	Pre10 - Post10	0.53	0.70	0.06	0.638	0.419	9.504	58	0.001

The qualitative comments reflected the pre-post quantitative changes on the individual questions. For example, the change on the statement “I am confident in my knowledge to support FCGs” was illustrated by this quote,

*I thought that this course was excellent. I was already aware of the importance of caregivers, but the content was simple, easy to understand, very practical and broadened my knowledge and confidence even more. (Registered Nurse)*

Similarly, the change seen on the statement “I know how to assist FCGs to navigate the system” was related to not wanting to ask about FCGs’ needs because of uncertainty about the availability of services to support them,

*I have always been worried about asking FCGs what they need because I don't know how to help them or what resources there are. I learned that I can ask my team. (Licenced Practical Nurse)*

### Changes in learner’s behavior in practice with family caregivers (level 3)

Three main themes emerged from the interviews that illustrated how the education impacted the 13 healthcare participants who had engaged in the education: (1) usable skills, (2) reinforced what I was doing, and (3) requires leadership and a change in culture.

#### Theme 1: usable skills

All interview participants referred to one or more elements of the education that they had used in practice.

*Really there isn't much education on how to care for like the patient's family or how to communicate with the family. When you are a student nurse you are nervous talking to the patient, no one talks about how to talk to the family. Then when you graduate, you might be lucky and see some who can talk easily with families, so I found that OARS communication framework so easy to use. (4*^*th*^*year student nurse)*

As this quote demonstrates, the most frequently mentioned skill was increased confidence to start conversations with FCGs due to the OARS verbal and non-verbal communication skills framework. OARS is a component of our Caregiver-Centered Care Education. “OARS” stands for **o**pen-ended questions, **a**ffirming, **r**eflective listening, and **s**ummarizing [77, 78]. OARS is a patient-centered approach designed to help healthcare providers engage and build rapport with caregivers by being a curious listener who wants to understand their situation, their needs, and their goals [77, 78]. Participants appreciated how easy it was to remember and noted that caregivers really appreciated the affirmation, that what they were doing was recognized. Easier conversations with caregivers enabled them to talk about the FCGs needs and signpost them to the resources they needed.

#### Theme 2: reinforced what I was doing

Many of these participants had been working with FCGs for some time. They welcomed the education because it reinforced good practices that they had established in interactions with FCGs. Many participants reported feeling that they had been alone in their commitment to supporting FCGs and welcomed the education to help others develop the skills and the will to support FCGs.

*It just reinforced what I was doing. I've shared it with many, many people. And many people have definitely appreciated the education and just learning where they stand or that we can be that person. (Primary care physician)*

#### Theme 3: requires leadership and change in culture

Aligned with the feeling that participants had been struggling alone to support caregivers, the third theme reinforced the need for support from healthcare leadership and a change in healthcare culture in order to provide caregivers with consistent support. Some learners credited leaders in their work setting with recognizing the importance of supporting caregivers to care and to maintain their own wellbeing, whereas other reported that their leadership was not supportive,

*This Caregiver-Centered Care Education was interesting. It should be mandatory for all areas of the health care treatment team. I just left a clinic with 2 physicians who do not include caregivers in any discussions and leave them in the waiting room. They do not want to even hear what the caregiver has to say, they say, “it’s not relevant.” (Nurse)*

These participants pointed out that there needed to be advocacy and policy changes to achieve a culture change that promoted the supporting of FCGs.

## Discussion

Researchers now recommend education to ensure healthcare providers are equipped with the competencies to support FCGs [11, 13, 27, 29, 79]. In this paper, we reported on a mixed methods evaluation of a competency-based education program for healthcare providers who interact and work with FCGs. The online delivery of the Foundational Caregiver-Centered Care Education program was acceptable to healthcare providers working in a range of settings including acute care, homecare, primary care, supportive living, and long-term care. The development of the curriculum and educational materials for our education for healthcare providers followed the Caregiver-Centered Care Competency Framework [52], which was created in consultation with international, national, and provincial multi-level interdisciplinary stakeholders and validated in a Modified Delphi process [38]. Multi-level, multidisciplinary stakeholders also co-designed the education content. Many different perspectives likely helped to make the content acceptable to a range of learners to ensure usable results that meet end-users needs [80].

The education program had an immediate effect of increasing learners’ knowledge and skills to work with FCGs, with 90.3% of learners reporting a knowledge gain at the end of the course. Learners became more confident and comfortable in supporting FCGs. Notably, the greatest self-rated improvements were in skills, such as communicating with FCGs, assisting FCGs to navigate health and community systems, and assessing family caregiver support needs. In qualitative interviews 6 months after the program, learners indicated that the communication skills were useful in practice and facilitated conversations with caregivers about their needs.

There are several novel findings about learning preferences, which may be useful for future development of education for healthcare providers. Learners were complementary about the value of realistic scenarios portrayed in the videos interspersed with reflective exercises delivered online. Several participants characterized it as “modern” education and appreciated that it took about an hour. Hospital nurses prefer shorter educational sessions [40, 81]. While we moved the education online to deal with the difficulty of offering face-to-face education during the COVID-19 pandemic, the education can be offered flexibly in shorter modules (online, face-to-face, blended learning) that can be customized to different clinical settings (primary care, homecare, acute care, supportive living, long-term care). We will be testing the impacts of customized delivery of the education in six short education sessions in long-term care settings in 2022. The interactive exercises will also be tailored to integrate reflection, critical thinking, and tools relevant to each setting.

Educating healthcare providers to provide person-centered care specifically for FCGs is a relatively rare undertaking. In their US study, Badovinac and colleagues [29] found that nurses and nursing leaders reported that working with FCGs is frequent and important, and also stated that Caregiver-Centered Care is not provided effectively. However, in much of the literature, ‘caregiver education’ has been interpreted to mean education for FCGs rather than education targeting healthcare providers who work with FCGs [13, 25, 29]. In this sense, the intervention we co-designed through extensive consultations with international and national experts, as well as stakeholders from a range of backgrounds [38], created an innovative education program to address a pressing issue.

Educating healthcare providers to support FCGs is one element towards addressing an inconsistent system of supports for FCGs. Given the heterogeneity of caregivers and their care situations (people, illnesses, settings), interventions must be tailored to FCGs’ specific needs and roles in their care trajectory [13, 43, 82, 83]. Health and social care providers’ resistance to adopt Caregiver-Centered Care [28, 29, 84] is further compounded by a lack of community awareness, limited resources, and policy deserts that create systemic barriers to supporting FCGs and addressing their needs [29, 36, 84]. We agree with Fields [85] that this may be a good time develop collaborative partnerships among care-receivers, FCGs, health and social care systems, and policy makers to build a coordinated system to support FCGs and the vulnerable people they care for.

### Limitations

One limitation on the study is that healthcare providers following the Foundational Caregiver-Centered Care Education program were likely self-selected people with an interest in supporting FCGs. While their evaluations were positive, the opinions of this sample may differ systematically from healthcare providers more generally. Furthermore, our education program has been designed in one province in Canada and thus may not be generalizable to other provinces or countries. However, there were as significant proportion of healthcare providers from Ontario and the education has been recommended nationally for staff education by Healthcare Excellence Canada. The goal is to evaluate the education with healthcare providers in other provinces and in specific healthcare settings.

Second, our study focused on the first three levels of the Kirkpatrick-Barr Framework. Healthcare education is rarely evaluated at levels three and four of the framework [57, 86]. We also had a relatively conservative sample to investigate Level 3. Nevertheless, our Foundational Caregiver-Centered Care Education program is a first step towards addressing calls in the literature [11, 27, 87] for the healthcare workforce to receive competency-based education that supports their capacity to identify, assess, support and partner with diverse FCGs throughout care trajectory. Third, we were not able to evaluate FCGs’ perspectives on how the Foundational Caregiver-Centered Care Education program impacted their interactions with healthcare providers who did versus did not complete the education. Our future research will seek to evaluate the impacts of education on FCGs’ perceptions of interactions with trained healthcare providers (level 4) and how learners utilize the material in practice (level 3).

Finally, this education is foundational, designed to educate all healthcare providers to take a person-centered approach to FCGs. While it did achieve that aim, it will not meet all learner’s needs, especially those who interact extensively with, or have significant leadership responsibilities for providers who work with FCGs. Specifically, four participants reported their high scores before and after the education reflected their experience working with FCGs. However, they still welcomed the training, even if they felt they had knowledge and skills. Unfortunately, we did not ask learners about their years of experience in healthcare in our quantitative data, which prevents sub-group comparison. We are developing advanced and champions modules to provide more in-depth training for providers who have more in-depth interactions with FCGs and we will ask about years of experience in future data collection.

## Conclusion

Given the increasing proportion of people needing care, as well as the increased responsibility for care being placed on FCGs by policies that move healthcare closer to home so people can age in place, FCGs need supports from healthcare providers. However, it is widely accepted that healthcare curricula do not include skills aimed to recognize the family caregiver’s role in the care trajectory, assess the family caregiver’s ability and needs to implement that care, include FCGs as partners in care, or assist FCGs to maintain their own wellbeing. Our Caregiver-Centered Care Education provides a foundation for educating healthcare providers to provide care to FCGs to maintain their own wellbeing and to help them continue to provide care to their care recipients. The education was rated as acceptable and shows significant promise in improving knowledge of and confidence in providing person-centered care to all FCGs. This is an important step towards shifting the culture of health care to include FCGs as partners in care.

## Supplementary Information

**Additional file 1.** Foundational Education: Key Learning Points.

**Additional file 2.** Interactive Exercises.

**Additional file 3.** Factor Matrix.

**Additional file 4.** Semi-structured Interview Guide Level 3.

**Additional file 5.** Table Stages of Thematic Analysis.

**Additional file 6.**

## Data Availability

The quantitative dataset supporting the conclusions of this article is included in Supplementary File 6: Excel File of Foundational Caregiver Centered Care Data. The qualitative data is available from the corresponding author.
