# Supplementary material for: Optimizing the integration of family caregivers in the delivery of person-centered care: evaluation of an educational program for the healthcare workforce
Source: BMC Health Serv Res. 2022 Mar 18;22:364. doi: 10.1186/s12913-022-07689-w (PMC8932680; doi:10.1186/s12913-022-07689-w)
Supplement: Supplementary file 1 — Additional file 1. Foundational Education: Key Learning Points. [file 12913_2022_7689_MOESM1_ESM.docx]

**Supplementary Materials 1 Foundational Education: Key Learning Points**

**Domain A: Recognizing the Caregiver Role**

- Family caregivers make tremendous contributions to the care recipient, healthcare system, and society.
- Negative impacts of family caregiving are primarily due to extensive demands of caregiving and limited supports.
- Family caregivers need to be identified and valued.

**Domain B: Communicating with Family Caregivers**

- Begin conversations by building rapport and demonstrating empathy.
- Affirm the family caregiver’s strengths and abilities.
- Tailor the type and amount of information to the family caregiver’s specific needs.

**Domain C: Partnering with Family Caregivers**

- There are several benefits to including family caregivers on the care team
- Partnering with caregivers is based on mutual appreciation, trust, and respect
- Caregivers are the experts in their caring roles and their knowledge can add to the quality of care provided to care receivers.

**Domain D: Fostering Resilience in Family Caregivers**

- Assessing family caregivers’ needs will ensure supports are tailored to the family caregiver’s needs.
- Recognize that like all relationships, there are 2 people in the caregiving/care recipient relationship.
- Self-care is the last thing that caregivers think they have time for.

**Domain E: Navigating Health and Social Systems and Accessing Resources**

- Help to navigate systems and resources makes the family caregiver’s care journey more manageable.
- Collaborate with family caregivers to identify and select the supports and services that are relevant for them.
- Share knowledge and the care plans with family caregivers and other health providers on the care team.

**Domain F: Enhancing the Culture and Context of Care.**

- We all have a role in promoting a culture of support for family caregivers in our workplaces.
- Modelling caregiver-centered care behaviours will create a culture that supports family caregivers.
- Reflecting on your practical experiences with caregivers will enable you to develop your caregiver-centered skills.
