# Supplementary material for: Optimizing the integration of family caregivers in the delivery of person-centered care: evaluation of an educational program for the healthcare workforce
Source: BMC Health Serv Res. 2022 Mar 18;22:364. doi: 10.1186/s12913-022-07689-w (PMC8932680; doi:10.1186/s12913-022-07689-w)
Supplement: Supplementary file 2 — Additional file 2. Interactive Exercises. [file 12913_2022_7689_MOESM2_ESM.docx]

**Supplementary Materials 2: Interactive Exercises**

**Family Caregiver's Pilot - Online Experience**

*Updated: April 29, 2020*

Guiding Design Principles:

Materials are self-paced (though learners should have the opportunity to post and see comments from others)

Estimated completion time - 60 mins (not including pre-and post-test quiz)

Goal is to educate learners (as opposed to assessing knowledge, skills, and attitudes)

Content needs to be actively engaged with

Learners should be given a specific time to complete the instruction

Note: Some groups may deliver the materials face-to-face and will need printable resources. The exact need is still to be determined.

| Welcome |
| --- |
| Thank you for being part of the pilot testing of Caregiver-Centered Care: Health Workforce Education to Support Family Caregivers.  This instruction will take about one hour to complete. But, before we begin, please complete this short pre-instruction assessment. |
| Session Setup (5 Minutes) |
| “There are only four kinds of people in the world. Those who have been caregivers. Those who are currently caregivers. Those who will be caregivers, and those who will need a caregiver.” ― Rosalyn Carter  Welcome to:  Foundational Caregiver-Centered Care: Health Workforce Education to Support Family Caregivers  Our goal is to introduce you to the importance of family caregivers and challenge you to think about how you can work with, and support family caregivers  The following competency framework for supporting family caregivers will be explored:  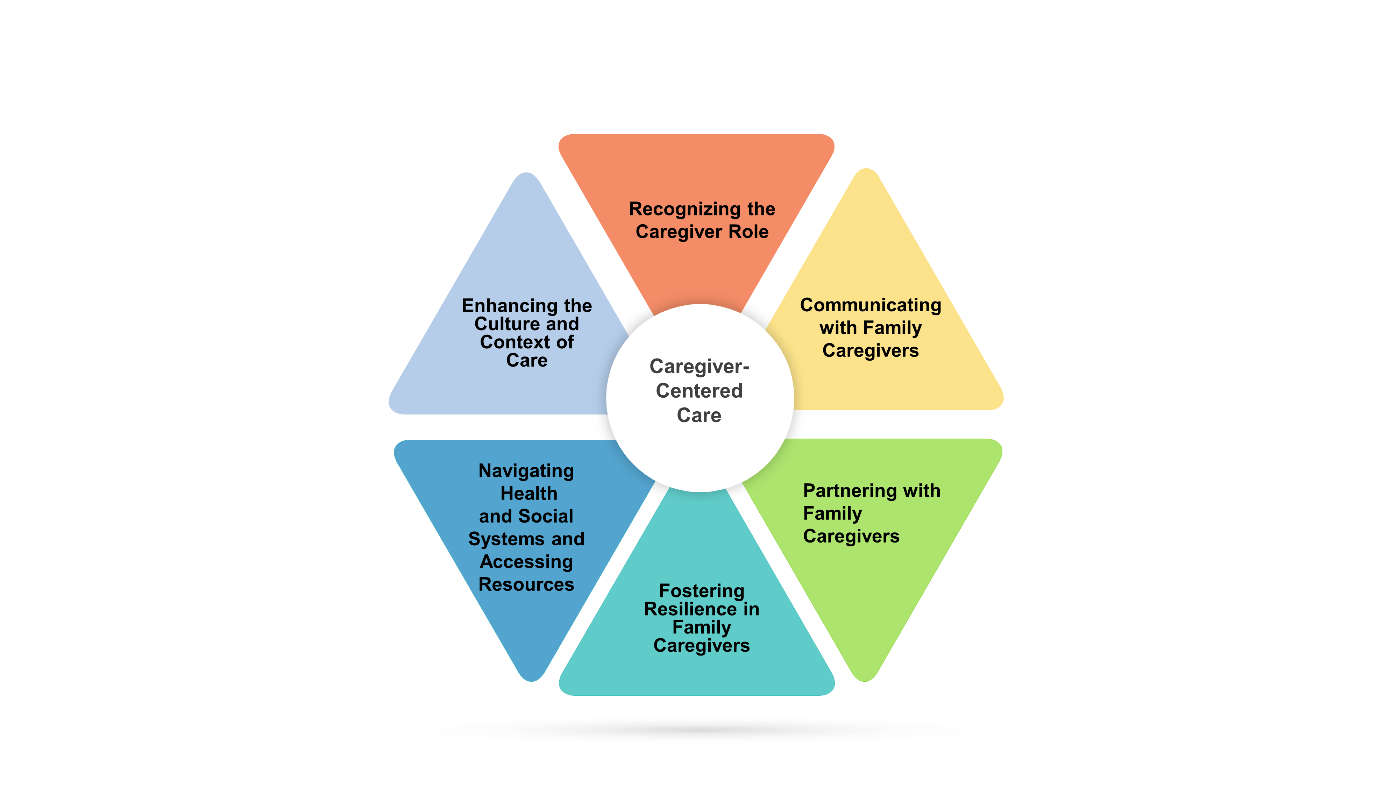  Approximate Course Time: One Hour  Course Activities: Videos, Reflections, and Surveys  Domain 1 Recognizing the family caregiver role  What percentage of Canadians are family caregivers? What is your estimate?  8%  17%  25%  34%  50%  Answer: Approximately 1 in 4 Canadians 15 and older provide care to care a family member or friend without pay (69, 70). (<https://www150.statcan.gc.ca/n1/en/pub/11-627-m/11-627-m2020001-eng.pdf?st=SgXX6CaD> OR <https://www150.statcan.gc.ca/n1/en/daily-quotidien/200108/dq200108a-eng.pdf?st=jIFabocF>  Think About  In your mind, picture a family caregiver that you know. It may be someone you know through your healthcare work, a personal contact, or it might be you.  Video #1 Domain 1: Recognizing the family caregiver role. |
| Video Presentation & Debrief - Recognizing the Family Caregiver Role (10 Minutes) |
| Post-video activity  In the video, Lacey and Davina are caregivers who are also health care providers. Do you think being a health care provider would make being a caregiver easier or harder?  The next video is about communication. Think of a conversation you had with a family caregiver that went particularly well.  Video #2 Domain 2: Communicating with Family Caregivers |
| Video Presentation & Debrief - Communicating with Family Caregivers (10 Minutes) |
| Post-video Activity  What communication skills did you use that made your conversation with the family caregiver go well?  After you have posted your experience, you will be able to see the experiences of others.  [We will need three default posts - not more than 3 sentences - for learners to view]  Note: Introducing myself to the caregiver. Asking questions to learn about the caregiver. Listening carefully to caregivers. Letting the caregiver what they did well.  Video #3 Domain 3: Partnering with Family Caregivers  Have you ever worked together with someone? That is teamwork or partnering. |
| Video Presentation & Debrief - Partnering with Family Caregivers (10 Minutes) |
| Post-video Activity  How do you think partnering with a family caregiver would help you in your role and workplace?  Video #4 Domain 4: Fostering Resilience in Family Caregivers  Key Question  Consider this key question as you watch Fostering Resilience  Has someone ever given you a present that you didn’t like? How did you feel? Understanding the needs of caregivers will help you to ensure they get what they need. |
| Video Presentation & Debrief - Fostering Resilience in Family Caregivers (10 Minutes) |
| Post-video Activity  Having a conversation with caregivers will help you to get to know them and their needs. If Lacey said to you, “I need to understand mom’s illness.” What question would you ask to understand what she needs to know about the illness?  Tip: Start by recognizing that what the caregiver wants to know is important, then ask a question that will help you to learn more….  Examples:  Yes, it is important to make sure we meet your mom’s needs. What concerns you the most?  There is so much to learn about this, Can you tell me what you are concerned about?  Video #5 Domain 5: Navigating the Health and Social Systems and Accessing Resources  Key Question  As you watch the video, think about what kinds of supports those family caregivers that you see in your workplace might need. |
| Video Presentation & Debrief - Navigating the Health and Social Systems and Accessing Resources (10 Minutes) |
| Try matching the caregiver’s needs with the resources listed  Understand dementia Pharmacist  I am stressed Homecare  I don’t understand the medications Alzheimer’s Society  Where can I get help to bathe him Family doctor/ Caregivers Alberta  Video #6 Domain 6: Enhancing the Culture and Context of Care  Role models are someone we look to as a good example. As you watch the video, who do you think is the best role model of supporting family caregivers? |
| Video Presentation & Debrief - Enhancing the Culture and Context of Care (10 Minutes) |
| Key Question  Who do you think is the best role model for supporting family caregivers?  What would you tell a co-worker about why you think that person is a good role model? |
| Thank You and Next Steps |
| Thank you for being part of the pilot testing of Caregiver-Centered Care: Health Workforce Education to Support Family Caregivers.  For information on Caregiver-Centered Care please visit our website at caregivercare.ca. If you wish to further education or assistance with family caregiver advocacy, please contact [contact information]  Before you leave, please be sure to complete this short post-instruction assessment.  Thank you again. |
