# Supplementary material for: Optimizing the integration of family caregivers in the delivery of person-centered care: evaluation of an educational program for the healthcare workforce
Source: BMC Health Serv Res. 2022 Mar 18;22:364. doi: 10.1186/s12913-022-07689-w (PMC8932680; doi:10.1186/s12913-022-07689-w)
Supplement: Supplementary file 3 — Additional file 3. Factor Matrix. [file 12913_2022_7689_MOESM3_ESM.docx]

**Supplementary Material 3: Factor Matrix**

| Factor Matrix^a^ | | |  |
| --- | --- | --- | --- |
|  | Factor | |  |
|  | 1 | 2 |  |
| I am comfortable in supporting family caregivers | 0.836 |  | |
| I know what it means to partner with family caregiver | 0.824 |  | |
| I know how to communicate with family caregivers. | 0.800 |  | |
| I am confident in my knowledge to support family caregivers | 0.785 | -0.359 | |
| I know my role in assessing caregiver needs | 0.776 |  | |
| I am aware of the contributions of family caregivers | 0.764 | 0.480 | |
| I understand the need to reflect on my interactions with family caregivers | 0.761 |  | |
| I know how to assist family caregivers to navigate the system | 0.756 | -0.332 | |
| I am comfortable in identifying family caregivers | 0.750 |  | |
| I am aware of the consequences of caring to family caregivers | 0.703 | 0.468 | |
| Extraction Method: Principal Axis Factoring. | | |  |
| a. 2 factors extracted. 9 iterations required. | | |  |
