# Supplementary material for: Optimizing the integration of family caregivers in the delivery of person-centered care: evaluation of an educational program for the healthcare workforce
Source: BMC Health Serv Res. 2022 Mar 18;22:364. doi: 10.1186/s12913-022-07689-w (PMC8932680; doi:10.1186/s12913-022-07689-w)
Supplement: Supplementary file 4 — Additional file 4. Semi-structured Interview Guide Level 3. [file 12913_2022_7689_MOESM4_ESM.docx]

**Supplementary Material 4: Semi-structured Interview Guide Level 3**

**Semi-structured Interview Guide on Caregiver-Centered Care Education for the Health Workforce and Trainees**

We are conducting these interviews to understand how you have used the Caregiver-Centered Care Education in your practice as well as how satisfied you were with the content of the education. Have you received the consent form and confidentially agreement? As we are doing this virtually on ZOOM, do I have your verbal consent to participate in the interview?

You can add my name______________________ and the date and time to your consent form.

***Experience working with family caregivers***

1. Can you tell us about your current work or education role?
2. Now can you tell us a bit your current work with family caregivers?

***Use in practice***

1. Have you been able to use what you learned in the education in practice?

Probes:

- 1. Can you describe your experience?
  2. Can you tell me a story about what you did before and how the education changed what you did?

1. Were there particular parts of the education that you found helpful in practice?

Probes:

1. Can you tell me how that helped you in your practice?
2. Can you give me an example?

- Recognizing the family caregiver role
- Communicating with family caregivers
- Partnering with family caregivers
- Fostering resilience in family caregivers
- Navigating health and social systems
- Enhancing the culture and context of care

***Satisfaction with content***

1. What was your overall impression of the Caregiver-Centered Care Education content?
2. Was the content of relevant for you?
   1. What would you suggest strengthening the content?
   2. What should be added?
   3. Did the scenarios resonate with your experience?
3. How did the scenarios presented in the videos resonate with you?

Probes:

In your experience as provider? family caregiver?

***Recommended Changes***

1. **What could we do better?**

***Last question***

1. We have asked you lots of questions, now it is your turn. Is there anything that you would like us to know?
2. Any questions for us?

Verbal Consent to interview.

Name of the person receiving verbal consent _____________________________________

Date of verbal consent _______________________________

Time of verbal consent________________________________

Name of person giving verbal consent: ___________________________________________
