# Supplementary material for: Optimizing the integration of family caregivers in the delivery of person-centered care: evaluation of an educational program for the healthcare workforce
Source: BMC Health Serv Res. 2022 Mar 18;22:364. doi: 10.1186/s12913-022-07689-w (PMC8932680; doi:10.1186/s12913-022-07689-w)
Supplement: Supplementary file 5 — Additional file 5. Table Stages of Thematic Analysis. [file 12913_2022_7689_MOESM5_ESM.docx]

**Supplementary Material 5: Table Stages of Thematic Analysis**

| Step 1 | Researchers familiarize themselves with data | Read transcripts and listen to the recordings. Make corrections in transcriptions, Make notes on tone, first impressions of the data. |
| --- | --- | --- |
| Step 2 | Identify preliminary codes | Identify interesting elements in data.  Document impressions with notes.  Write memos on emerging themes.  Document connected elements. |
| Step 3 | Find themes in the data | Look for and identify themes in the data.  Identify all data relevant to the themes. |
| Step 4 | Finalize the themes | Finalize the themes.  Check for data overlaps between the themes. |
| Step 5 | Review each theme | Review the data to ensure that data fits each unique theme. Reread the transcriptions to ensure the overall data fits with the overall storyline and each theme. |
| Step 6 | Document and review documented analysis | Analyze the resultant documentation and the inferences drawn. |
